# Molecular and Functional Characterization of cDNAs Putatively Encoding Carboxylesterases from the Migratory Locust, *Locusta migratoria*

**DOI:** 10.1371/journal.pone.0094809

**Published:** 2014-04-10

**Authors:** Jianqin Zhang, Daqi Li, Pingting Ge, Yaping Guo, Kun Yan Zhu, Enbo Ma, Jianzhen Zhang

**Affiliations:** 1 Research Institute of Applied Biology, Shanxi University, Taiyuan, Shanxi, People's Republic of China; 2 Department of Entomology, Kansas State University, Manhattan, Kansas, United States of America; Natural Resources Canada, Canada

## Abstract

Carboxylesterases (CarEs) belong to a superfamily of metabolic enzymes encoded by a number of genes and are widely distributed in microbes, plants and animals including insects. These enzymes play important roles in detoxification of insecticides and other xenobiotics, degradation of pheromones, regulation of neurodevelopment, and control of animal development. In this study, we characterized a total of 39 full-length cDNAs putatively encoding different CarEs from the migratory locust, *Locusta migratoria*, one of the most severe insect pests in many regions of the world, and evaluated the role of four CarE genes in insecticide detoxification. Our phylogenetic analysis grouped the 39 CarEs into five different clades including 20 CarEs in clade A, 3 in D, 13 in E, 1 in F and 2 in I. Four CarE genes (*LmCesA3*, *LmCesA20*, *LmCesD1*, *LmCesE1*), representing three different clades (A, D and E), were selected for further analyses. The transcripts of the four genes were detectable in all the developmental stages and tissues examined. *LmCesA3* and *LmCesE1* were mainly expressed in the fat bodies and Malpighian tubules, whereas *LmCesA*20 and *LmCesD1* were predominately expressed in the muscles and hemolymph, respectively. The injection of double-stranded RNA (dsRNA) synthesized from each of the four CarE genes followed by the bioassay with each of four insecticides (chlorpyrifos, malathion, carbaryl and deltamethrin) increased the nymphal mortalities by 37.2 and 28.4% in response to malathion after *LmCesA20* and *LmCesE1* were silenced, respectively. Thus, we proposed that both *LmCesA20* and *LmCesE1* played an important role in detoxification of malathion in the locust. These results are expected to help researchers reveal the characteristics of diverse CarEs and assess the risk of insecticide resistance conferred by CarEs in the locust and other insect species.

## Introduction

Carboxylesterases (CarEs, EC 3.1.1.1) belong to a superfamily of metabolic enzymes and are widely found in microbes, plants, and animals including insects [1], [2], [3]. Various schemes have been used to classify different eukaryotic CarEs, including the sensitivity to different inhibitors [4], isozymic variations and thermostability [5], and DNA or protein sequence similarity [6]. Mammalian CarEs (commonly abbreviated as CES) have been classified into six main groups and several additional subgroups based on their amino acid sequence homology and substrate specificity [6]. Based on the same criteria, insect carboxyl/cholinesterases can be classified into 14 major clades (denoted from A to N) and fall into three functional groups: dietary detoxification (A-C), hormone and pheromone degradation (D-G), and neurodevelopmental functions (H-N) [4], [7].

The major attributes of some members of dietary detoxification group include those involved in the metabolism of insecticides in the diet or from the environment. For example, a cytosolic esterase (AAC36246) from clade A has been found to be responsible for high level of malathion resistance in *Anisopteromalus calandrae* due to a point mutation [8]. Members of the second group (clades D-G) are virtually all secreted enzymes, which include pheromone degrading esterases (PDEs), juvenile hormone (JH) esterases (JHEs) and β-esterases [4]. PDEs are mainly expressed in male antennae and play an important role in the dynamics of the olfactory response to acetate sex pheromones in insects [9]. JHEs degrade JH in hemolymph and play a key role in the regulation of various physiological processes [10], [11]. Some members of β-esterases located in clade E are known to confer organophosphate (OP) and carbamate resistance by overexpression of the genes in several hemipteran species [12], [13]. The third group, except for acetylcholinesterases (AChEs) that hydrolyze the neurotransmitter acetylcholine, contains non-catalytic proteins with essential functions in neurodevelopment [4].

**Figure 1:**
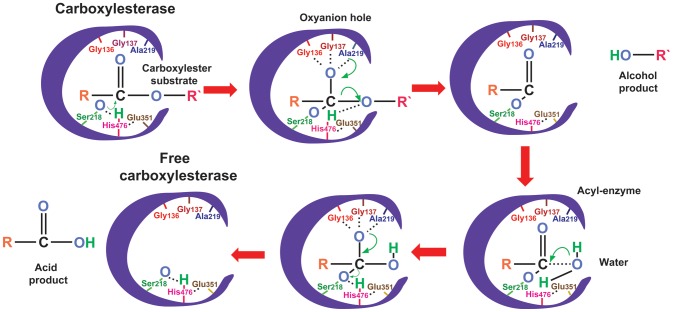
A brief schematic illustration of the biochemical reaction catalyzed by a carboxylesterase. Two-step hydrolysis reaction was described as follows: In the first step, the oxygen of a serine residue in the active site makes a nucleophilic attack on the carbonyl carbon of the substrate, releasing the alcohol product and forming a relatively stable acyl-enzyme. In the second step water makes a similar nucleophilic attack, releasing acid product and regenerate the free carboxylesterase. The diagrammatic representation is based on Oakeshott et al. (2005). Each residue is followed by its position in the sequence of DmJHE (accession number: NP_523758.3). The oxyanion hole is formed by Gly136, Gly137 and Ala219, and the catalytic triad is formed by Ser218, Glu351 and His476 in DmJHE.

Because the primary biological function of CarEs is to hydrolyze various carboxylic esters to alcohols and acids (Fig.1) [4], CarEs play a major role in conferring resistance to insecticides, such as OPs, carbamates and pyrethroids, through gene amplification, mRNA upregulation and amino acid substitution in insects [14]-[16]. The first crystal structure of an insect CarE (LcαE7) was recently reported in the Australian sheep blowfly, *Lucilia cuprina* [17], which represents as one of the best studies on CarEs involved in insecticide resistance. To date, the genome sequence analyses have revealed a number of CarE genes in 10 model species of insects belonging to five different orders. These orders include Diptera (35 genes in *Drosophila melanogaster* [4], 51 in *Anopheles gambiae* [18], 56 in *Aedes aegypti* [19], and 71 in *Culex pipiens quinquefasciatus* [20]), Lepidoptera (69 in *Bombyx mori* [21]), Hymenoptera (24 in *Apis mellifera* [22], 41 in *Nasonia vitripennis* [6]), Coleoptera (49 in *Tribolium castaneum*) [23], and Hemiptera (28 in *Acyrthosiphon pisum* [24] and 24 in *Trialeurodes vaporariorum* [25]). However, only a few CarE genes have been revealed for their detoxification function in insects.

The migratory locust, *Locusta migratoria* (Orthoptera: Acridoidea), is a serious insect pest of prairies and croplands. Destructive outbreaks of locust are increasing in the recent years in Tibet of China owing to the suitable ecological environment [26]. Extensive applications of OPs to control the locust have resulted in the development of resistance in some field populations [27]–[30]. The identification of insecticide resistance in field populations has prompted our research to reveal the function of various detoxification genes in the locust. The availability of the locust EST database (http://locustdb.genomics.org.cn) [31] has recently accelerated the pace of such researches in the locust.

In this study, we identified and characterized 39 full-length cDNA sequences of CarE genes from the locust. We studied the developmental and tissue-specific expression patterns of four representative genes (*LmCesA3*, *LmCesA20*, *LmCesD1* and *LmCesE1*) from three different clades. Finally we further performed RNA interference (RNAi) for each of the four genes followed by insecticide bioassay to reveal the role of these genes in detoxification of four commonly used insecticides in the locust.

## Materials and Methods

### Insect

The migratory locusts were procured from the Insect Protein Co., Ltd. of Guangzhou City in China. The insects were reared in plastic cages (22×22×22 cm) at 28±1°C under 14∶10 h (light: dark) cycles for one generation.

### Identification and Phylogenetic Analysis of *L. migratoria* CarE Genes

The cDNA sequences of CarE genes were searched using the keywords including carboxylesterase, acetylcholinesterase, neuroligins, glutactin, gliotactin and neurotactin against the locust transcriptome database that was developed based on the sequences from many samples collected from different developmental stages (egg, five nymphal instars and adult). The homology searches were performed through BLASTx against the NCBI non-redundant (nr) database using the *E*-value cut-off of 1E^−10^. The cDNA sequences of the candidate genes were then assembled by Sequencher software 4.14 (http://www.sequencher.com/) and the selected candidate genes of a minimum length of 500 nucleotides were used in our analyses. BLASTx searches with the *E*-value cut-off of 1E^−17^ were then performed against the NCBI non-redundant (nr) database. The predicted polypeptides with at least one of the conserved motifs (Ser218, Glu351, His476, Gly136Gly137Gly138 or N-terminal conserved Cys113 for disulfide bond) were selected for further analyses. The number followed by each conserved residue indicates its position in the sequence of DmJHE (GenBank accession No. CG8424).

To construct a phylogenetic tree for the locust CarEs, we included most of the insect CarEs that were used by Oakeshott et al. [4] in our analysis. Our purpose was to classify the locust CarEs based on the classification scheme for insect CarEs as previously established [4]. Briefly, the deduced amino acid sequences of the locust CarE cDNAs were aligned with other insect CarEs by ClustalW software [32]. MEGA5.0 was then used to construct a phylogenetic tree using the neighbor-joining method [33]. The bootstrap analysis of 1000 replicates was performed to evaluate branch strength in the phylogenetic tree. The locust CarEs were named based upon the major clades with which they were clustered. For example, LmCesA3 represents the third CarE in clade A from the locust.

### Analyses of Deduced Amino Acid Sequences of *L. migratoria* CarE Genes

The amino acid sequences of the locust CarE cDNAs were deduced by using the ExPASy Proteomics programs (http://cn.expasy.org/) [34]. The molecular mass (MM) and isoelectric point (pI) for each deduced CarE were predicted by using ExPASy tools (http://www.expasy.ch/). To identify the catalytic triad presented in deduced amino acid sequences, BLASTp analyses were undertaken based on the NCBI Conserved Domain Database (http://www.ncbi.nlm.nih.gov/cdd). Signal peptide for each protein was predicted by using SignalP4.0 web tools (http://www.cbs.dtu.dk/services/signalp/). The NetNGlyc1.0 Server (http://www.cbs.dtu.dk/services/NetNGlyc/) was used to predict potential *N*-glycosylation sites of locust CarEs. Finally, the locust and other insect CarEs located in same clade were aligned by using GeneDoc software (http://www.nrbsc.org/gfx/genedoc/).

### Subcloning and Sequencing of Four Selected *L. migratoria* CarE Genes

Because some members of insect CarEs located in clades A, D and E have been known to be involved in insecticides resistance in other insects, we selected four CarE genes (*LmCesA3*, *LmCesA20*, *LmCesD1*, *LmCesE1*) from these clades for further studies. Our transcriptome data have already revealed complete ORFs of these CarE genes; therefore, we just needed to confirm each sequence by amplifying its complete ORF using a pair of gene-specific primers and the first-strand 5′-RACE Ready cDNA as a template. The use of the first-strand 5′-RACE Ready cDNA as a template was to increase the possibility of amplifying a complete ORF by regular PCR. In brief, total RNA was isolated from the pool of the whole bodies of six fifth-instar nymphs by using TRIzol Plus reagent (Takara, Dalian, China). The mRNA was isolated using PolyATtract mRNA isolation systems (Promega, Madison, WI, USA). After the first-strand 5′-RACE Ready cDNA was synthesized from 1 µg mRNA using Smart RACE cDNA Amplification kit (Clontech, Mountain View, CA, USA), regular PCR was performed using Advantage 2 Polymerase (Clontech) and primers as shown in Table 1. The PCR product from each reaction was subcloned into pGEM-T easy Vector (Promega) and sequenced using Applied Biosystems 3730XL by Beijing AuGCT Biotechnology Company (Beijing, China).

**Table 1:** Primer sequences and predicted sizes of PCR products for full-length cDNA cloning, RT-qPCR analysis and dsRNA synthesis of four *LmCarE* genes along with those in dsRNA synthesis for the control gene (*GFP*) and the reference gene (*β-actin*) in RT-qPCR analysis.

Gene name	Application of primers	Sequences (5′-3′)*	Product size (bp)
*LmCesA3*	Full-length cDNA amplification	F: GCGGAGCGACATGTCTTCC	1738
		R: ATGCTGCTCTTTTTTAGTGAGCATT	
	RT-qPCR analysis	F: ACGACACCTTCCAGCAGTTCG	164
		R: TCCGAACATCATTTTGAACAGGTT	
	dsRNA synthesis	F: taatacgactcactatagggCAACATGGGACTCAAAGACCAAGTT	562
		R: taatacgactcactatagggATGCCAGCGTAGAGGTAGCCTT	
*LmCesA20*	Full-length cDNA amplification	F: GCCATGAATCCGTGCCTTCTCCA	1712
		R: GCGACCTCTTTAACGTACAG	
	RT-qPCR analysis	F: GAGGAGCGCATGGCATTC	75
		R: TGCGACCTCTTTAACGTACAGACT	
	dsRNA synthesis	F: taatacgactcactatagggATTCGTGACAAATCGCAAATG	590
		R: taatacgactcactatagggCCCTGCCTATGTCCAGGAAGT	
*LmCesD1*	Full-length cDNA amplification	F: TGCTGGGATGTCACGGTCTC	1668
		R: GTAAAGCACTAATACTAATGAACCA	
	RT-qPCR analysis	F: CAGACTTCTGAAGACTGCTTGTTTCTA	105
		R: GGCACCAGGATGTAAGAAGATCA	
	dsRNA synthesis	F: taatacgactcactatagggGAAGAAATTGTTGAATGCCTGAAAG	532
		R: taatacgactcactatagggTGTACCTTCCTGCATATGTGAAGTG	
*LmCesE1*	Full-length cDNA amplification	F: GAAGATTTGGTGAGGTGAACAGTG	1796
		R: TTGTTAGGCATAATCCGTTTAGAGA	
	RT-qPCR analysis	F: ACTGCCTGAGGAGCGTGGAT	121
		R: AATGATTCTCTTCCTTCACCTTCC	
	dsRNA synthesis	F: taatacgactcactatagggCTTCACTCCTCCAATTCCTTTTC	523
		R: taatacgactcactatagggGGATGTTGGATAGTAAGAGTTGTTCG	
*GFP*	dsRNA synthesis	F: taatacgactcactatagggGTGGAGAGGGTGAAGG	712
		R: taatacgactcactatagggGGGCAGATTGTGTGGAC	
*β-actin*	RT-qPCR analysis	F: CGAAGCACAGTCAAAGAGAGGTA	156
		R: GCTTCAGTCAAGAGAACAGGATG	

*The lowercase letters represent the T7 promoter sequences for efficient in vitro transcription in dsRNA synthesis.

### Analyses of Tissue- and Stage-Dependent Expression of CarE Genes

For tissue-dependent expression studies, total RNA isolated from each of nine tissues, including the brain, foregut, gastric caeca, midgut, Malpighian tubules, hindgut, fat bodies, muscles and hemolymph from fifth-instar nymphs (3-day-old) was used. For stage-dependent expression studies, 19 samples of different developmental stage and time combinations, including the egg and three developmental times (day 1, 3 and 5) of other six developmental stages (the five nymphal instars and adult stage), were collected. Total RNA was isolated from the whole bodies of each sample by using TRIzol Plus reagent (Takara). Ten eggs or at least 3 nymphs or adults were pooled as a sample for total RNA preparation. The first strand cDNA was synthesized from 4 µg of total RNA by using oligo (dT)^18^ primer and RevertAid H Minnus M-Mulv reverse transcriptase (Fermentas, Glen Burnie, MD, USA).

The relative expression level of each CarE gene was determined in different tissues or developmental stages by reverse transcription quantitative PCR (RT-qPCR) using *β-actin* as a reference gene. Our previous studies showed that *β-actin* was a suitable reference gene for normalization of our expression data in different tissues or developmental stages of the locust [35]. The primers and the expected size of each PCR product are summarized in Table 1. RT-qPCR was performed with three biological replications (each with three technical replications) on ABI 7300 Real-Time PCR detection system (Applied Biosystems Inc., Foster City, CA, USA). The 20-µL PCR reaction mixture consisted of 0.8 µL of 0.4 µM of each primer, 10 µL SYBR Green Real-time PCR Master Mix (TOYABO, Japan), 2 µL of 20-fold diluted template cDNA and 6.4 µL of deionized water. The PCR cycling parameters included an initial denaturation at 95°C for 60 s, followed by 40 cycles of 95°C for 15 s, 60°C for 15 s and 72°C for 45 s, and a final step of dissociation that was automatically added by ABI 7300 Real-Time PCR detection system.

Relative expression was determined by using the double standard curve method [36]. Briefly, standard curves for both the target gene (CarE) and the reference gene (*β-actin*) were separately generated based on the log concentrations of samples in serial dilutions against their corresponding threshold cycle (Ct) values obtained from RT-qPCR. The standard curves were then used to extrapolate relative expression results for both the target and reference genes in experimental samples. After the relative expression result for the target gene was normalized to that of the reference gene in the same sample, the normalized results of the target gene were compared between the treatment and the control. Results were presented as mean ± standard errors. The statistical differences among the samples were determined by using Fisher's least significance difference (LSD) method (*P*<0.05).

### Evaluation of Detoxification Function of Four Selected CarE Genes

To assess possible roles of CarE genes in insecticide detoxification in the locust, RNAi followed by insecticide bioassay was performed as previously described [37]. Briefly, we used E–RNAi (http://www.dkfz.de/signaling/e-rnai3/) to design the primers that were used to synthesize double-stranded RNA (dsRNA). Because the locust genome is not available at this point, complete avoidances of significant homologies between the regions used in our dsRNA primer designs and other genes were not feasible. However, we rechecked each primer pair for potential cross-amplifications of other locust CarE genes by using Primer Premier 5.0 software. A cDNA region with <50% nucleotide identity with other locust CarE cDNAs was finally used to synthesize the primers for dsRNA synthesis (Table 1). PCR was performed by using the following protocol: 94°C for 60 s; 5 cycles of 94°C for 30 s, 68°C for 30 s and 72°C for 40 s; 35 cycles of 94°C for 30 s, 60°C for 30 s and 72°C for 40 s; and a final extension of 72°C for 10 min. PCR products were examined on 1.5% agarose gel and purified with Wizard SV Gel and PCR Clean-Up System (Promega). About 2 µg of each purified PCR product was used to synthesize dsRNA by using T7 RiboMAX Express RNAi System (Promega). The dsRNA was dissolved in deionized water, and the final concentration of each dsRNA was adjusted to 1.5 µg/µL.

To perform RNAi, 3 µg of the dsRNA synthesized from each of the four selected CarE genes (ds*LmCes*, treatment) or the dsRNA of the green fluorescent protein (GFP) gene (ds*GFP*, control) was injected into the abdomen between the second and third abdominal segments of each second-instar nymph (2-day-old) by using a microsyringe as previously described [37]. After the injection, the nymphs were reared in a growth chamber at 28±1°C with the photoperiod of 14∶10 h (light∶dark) and the humidity of 50%.

For each insecticide bioassay, 33–45 second-instar nymphs from the treatment or control group at 24 h after the dsRNA injection were randomly divided into three subgroups (each with 11–15 insects as a replication), and each nymph was topically applied with a 3-µL droplet of an insecticide solution (240 ng for malathion, 15 ng for chlorpyrifos, 51 ng for carbaryl and 0.6 ng for deltamethrin) onto the abdomen between the second and third sterna. These insecticide doses were predetermined to be approximately LD_30_ by bioassays. Mortality was recorded at 24 h after the insecticide treatments.

To assess the efficiency of RNAi, nymphs were collected for analyzing the transcript levels of each target gene at 12, 24 and 48 h after the dsRNA injections. A total of nine nymphs from the ds*GFP*-injected group (control) or the ds*LmCes*-injected group (treatment) were randomly separated into three subgroups (three insects per subgroup). Each subgroup collected at each of the three time points was used to isolate total RNA for RT-qPCR analyses as described above. Relative transcript levels were determined by using the 2^−ΔΔCt^ method [38] with*β-actin* as a reference gene. The amplification efficiency of each gene was estimated from the slope given by different amplifications from the cDNA sample with the 1∶10 serial dilutions according to the equation *E* = 10^[−1/slope]^ [38].

## Results

### Identification and Phylogenetic Analysis of CarE Genes

**Figure 2:**
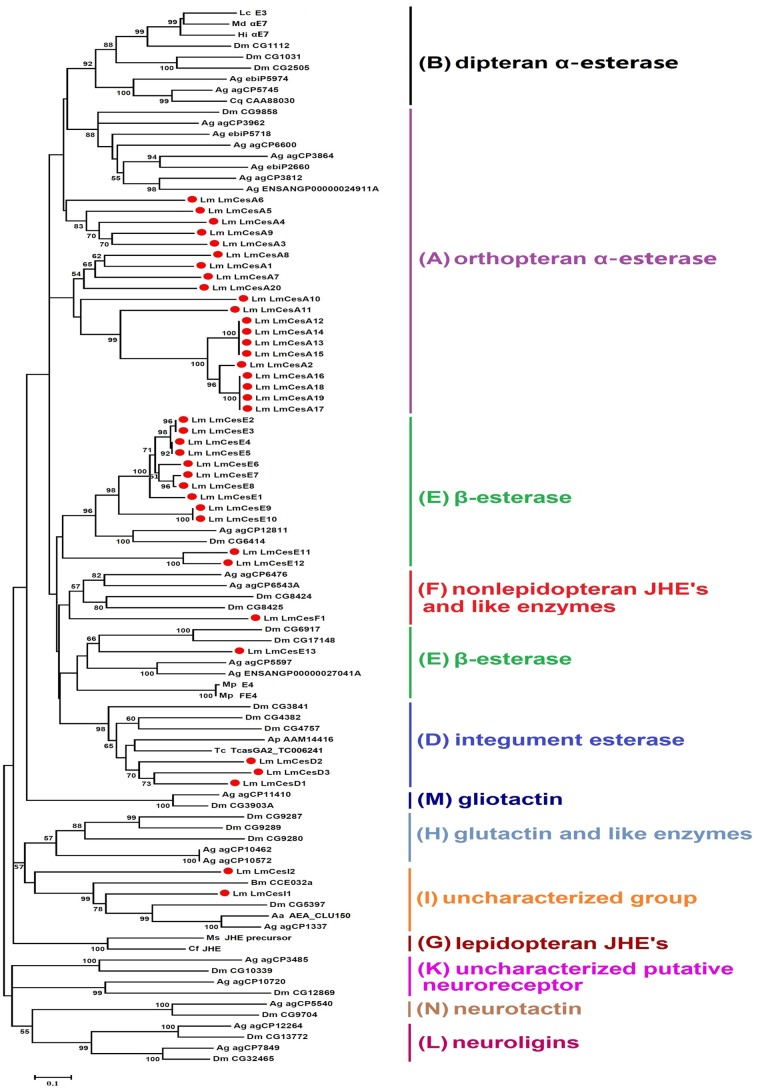
Phylogenetic analyses of insect carboxylesterases (CarEs). MEGA 5 was used to construct the phylogenetic tree with neighbor-joining method. Nodes with distance bootstrap values greater than 50% (1000 replicates) are shown. The nomenclatures of the clades are according to Oakeshott et al. [4]. Sequences from the *D. melanogaster* and *A. gambiae* genomes are annotated as in Ranson et al. [71].The 39 deduced CarEs from *L. migratoria* are marked with filled circles. The GenBank accession numbers for various CarEs of insects used in this tree were generally taken from NCBI database (http://www.ncbi.nlm.nih.gov/). The tree included twelve clades named A-N (except for C and J clade). The abbreviations used for insect species are: *Drosophila melanogaster* (Dm), *Anopheles gambiae* (Ag), *Culex quinquefasciatus* (Cq), *Aedes aegypti* (Aa), *Lucilia cuprina* (Lc), *Haematobia irritans* (Hi), *Musca domestica* (Md), *Myzus persicae* (Mp), *Antheraea polyphemus* (Ap), *Manduca sexta* (Ms), *Choristoneura fumiferana* (Cf), *Bombyx mori* (Bm), and *Tribolium castaneum* (Tc).

We identified a total of 84 putative CarE cDNA fragments from *L. migratoria* transcriptome. The deduced amino acid sequences of these fragments contain at least one of the conserved sequence motifs including S, E and H for catalytic triad, GGG for substrate binding pocket, and N-terminal conserved Cys for a disulfide bond. Among the 84 putative CarE cDNAs, 39 were full-length and contained complete ORFs. Figure 2 shows the phylogenetic tree constructed based on the 39 locust CarEs along with 57 CarEs from other insect species including *Anopheles gambiae*, *Drosophila melanogaster*, *Culex quinquefasciatus*, *Aedes aegypti*, *Lucilia cuprina*, *Haematobia irritans*, *Musca domestica*, *Myzus persicae*, *Antheraea polyphemus*, *Manduca sexta*, *Choristoneura fumiferana*, *Bombyx mori* and *Tribolium castaneum.* These insect species represent a total of five taxonomic orders (Orthoptera, Diptera, Hemiptera, Lepidoptera and Coleoptera). The clades of the *L. migratoria* CarE phylogeny were designated according to Oakeshott et al. [4], and the gene accession numbers of other insect CarEs are shown in Table 2. A different phylogenetic tree was also constructed with 71 locust CarEs including the 39 with complete ORFs and 32 partial sequences but with high similarities to insect CarEs (Fig. S1). In both the phylogenetic trees, a total of 12 clades representing three groups, including dietary/detoxification, hormone/semiochemical processing, and neuro/developmental functions, were formed. Our 39 locust CarEs were distributed in five clades, including clade A with orthopteran α-esterases (20 CarEs), clade D with integumental esterases (3 CarEs), clade E with β-esterases (13 CarEs), clade F with nonlepidopteran JHEs (1 CarE) and clade I with uncharacterized esterases (2 CarEs) (Fig. 2, Table 3).

**Table 2:** Accession numbers of other insect carboxylesterases used in phylogenetic analysis.

Clade	Gene name used in figure 1	Insect order	Species	Accession number
B	agCP3962	Diptera	*Anopheles gambiae*	XP_309020.3
	ebiP5718	Diptera	*Anopheles gambiae*	XP_309019.4
	ebiP2660	Diptera	*Anopheles gambiae*	XP_316734.4
	agCP6600	Diptera	*Anopheles gambiae*	XP_315770.4
	agCP3812	Diptera	*Anopheles gambiae*	XP_309018.4
	agCP3864	Diptera	*Anopheles gambiae*	XP_309021.4
	ENSANGP00000024911A	Diptera	*Anopheles gambiae*	XP_309017.3
	CG9858	Diptera	*Drosophila melanogaster*	NP_536784.1
	CG1031	Diptera	*Drosophila melanogaster*	AAF54002
	CG2505	Diptera	*Drosophila melanogaster*	AAF54003.2
	CG1112	Diptera	*Drosophila melanogaster*	AAF54010
	aE7	Diptera	*Musca domestica*	AF133341
	E3	Diptera	*Lucilia cuprina*	AAB67728
	aE7	Diptera	*Haematobia irritans*	AF139082
	ebiP5974	Diptera	*Anopheles gambiae*	XP_316296.3
	agCP5745	Diptera	*Anopheles gambiae*	XP_316295.4
	CAA88030	Diptera	*Culex quinquefasciatus*	CAA88030
D	CG3841A	Diptera	*Drosophila melanogaster*	ADV37009
	CG4382	Diptera	*Drosophila melanogaster*	AAF52792
	CG4757	Diptera	*Drosophila melanogaster*	AAF54592
	AAM14416	Lepidoptera	*Antheraea polyphemus*	AAM14416
	TcasGA2_TC006241	Coleoptera	*Tribolium castaneum*	EFA08586
E	agCP12811	Diptera	*Anopheles gambiae*	XP_322067
	agCP5597	Diptera	*Anopheles gambiae*	XP_556011
	ENSANGP00000027041A	Diptera	*Anopheles gambiae*	XP_556009.2
	CG6414	Diptera	*Drosophila melanogaster*	AAF45912
	CG6917	Diptera	*Drosophila melanogaster*	AAF49946
	CG17148	Diptera	*Drosophila melanogaster*	AAF49945
	E4	Hemiptera	*Myzus persicae*	P35501
	FE4	Hemiptera	*Myzus persicae*	P35502
F	agCP6476	Diptera	*Anopheles gambiae*	XP_315860.3
	agCP6543A	Diptera	*Anopheles gambiae*	XP_315859.3
	CG8424	Diptera	*Drosophila melanogaster*	NP_611085.2
	CG8425	Diptera	*Drosophila melanogaster*	NP_523758.3
G	JHE precursor	Lepidoptera	*Manduca sexta*	AF327882;
	JHE	Lepidoptera	*Choristoneura fumiferana*	AF153367
H	agCP10462	Diptera	*Anopheles gambiae*	XP_309784.4
	agCP10572	Diptera	*Anopheles gambiae*	agCP10572
	CG9287	Diptera	*Drosophila melanogaster*	NP_609244.1
	CG9289	Diptera	*Drosophila melanogaster*	ADO51073.1
	CG9280A	Diptera	*Drosophila melanogaster*	NP_477504.1
I	agCP1337	Diptera	*Anopheles gambiae*	agCP1337
	CG5397	Diptera	*Drosophila melanogaster*	AAF51389.1
	AEA_CLU150	Diptera	*Aedes aegypti*	AF466590
	NP_001040411	Lepidoptera	*Bombyx mori*	NP_001040411
K	agCP3485	Diptera	*Anopheles gambiae*	EAA04720.3
	agCP10720	Diptera	*Anopheles gambiae*	EAA07194.4
	CG10339	Diptera	*Drosophila melanogaster*	AAF47159.1
	CG12869	Diptera	*Drosophila melanogaster*	AAF58235.2
L	agCP12264	Diptera	*Anopheles gambiae*	EAA01441.5
	agCP7849	Diptera	*Anopheles gambiae*	XP_313315.2
	CG13772	Diptera	*Drosophila melanogaster*	NP_523496.1
	CG32465	Diptera	*Drosophila melanogaster*	NP_731170.2
M	agCP11410	Diptera	*Anopheles gambiae*	XP_317277.4
	CG3903A	Diptera	*Drosophila melanogaster*	AAF54480
N	agCP5540	Diptera	*Anopheles gambiae*	XP_315662.4

**Table 3:** Comparison of carboxylesterase numbers identified among *Locusta migratoria* (Orthoptera), *Drosophila melanogaster* (Diptera), *Anopheles gambiae* (Diptera), *Culex pipiens quinquefasciatus* (Diptera) and *Trialeurodes vaporariorum* (Hemiptera).

Carboxylesterase groups	*L. migratoria*	*D. melanogaster*	*A. gambiae*	*C. p. quinquefasciatus*	*T. vaporariorum*
**Dietary detoxification group**					
(A) orthopteran α-esterases	20	0	0	0	0
(B) dipteran α-esterases	0	13	16	30	12
**Hormone/semiochemical processing**					
(D) integument esterase	3	3	0	1	0
(E) β-esterase	13	3	5	3	6
(F) nonlepidopteran JHE's	1	2	5	13	0
(G) lepidopteran JHE's	0	0	4	9	0
**Neuro/developmental functions**					
(H) glutactin	0	4	9	6	1
(I) uncharacterized	2	1	1	1	1
(K) uncharacterized, putative neuroreceptors	0	2	2	0	0
(L) neuroligins	0	4	5	3	3
(M) gliotactins	0	1	1	1	1
(N) neurotactins	0	1	1	2	1
**Total number of carboxylesterases**	**39**	**34**	**49**	**69**	**25**

Classifications of different carboxylesterases were based on Oakeshott et al. [4] and Karatolos et al. [25].

### Sequence Comparison of *L. migratoria* CarEs

**Figure 3:**
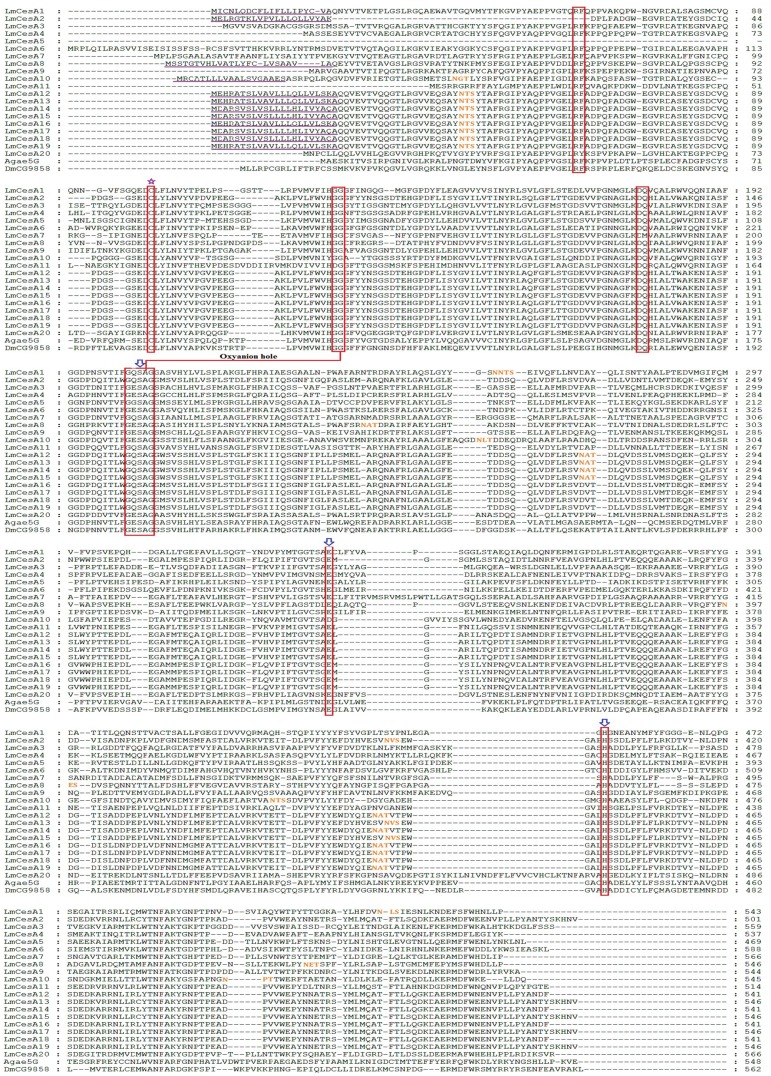
Alignment of deduced amino acid sequences of 20 *L. migratoria* CarE genes along with Agae5G and DmCG9858 in clade A. The multiple sequence alignment was performed using GeneDoc software. The predicted signal peptide is underlined in purple, the conserved motifs are boxed in red, N-terminal conserved Cys for disulfide bond is marked with purple star at the top, residues for oxyanion hole are linked with red line, the catalytic triads are marked with blue arrows at the top, and potential *N*-glycosylation sites are in orange.

**Figure 4:**
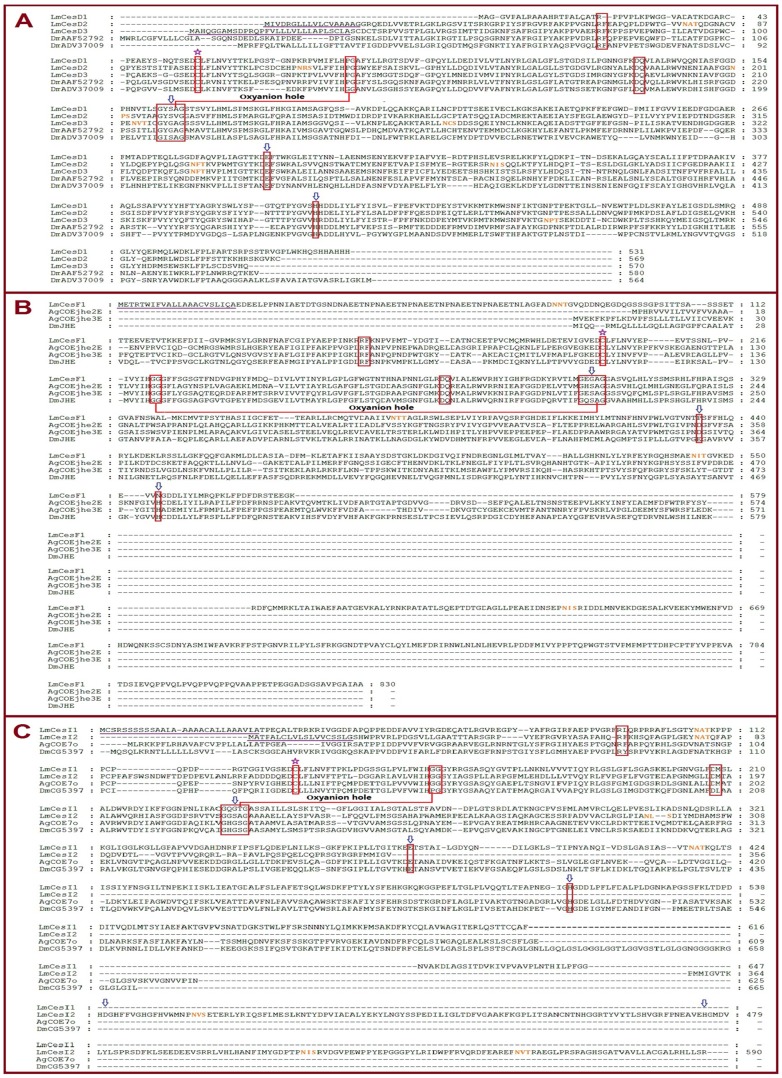
Alignment of deduced amino acid sequences of selected *L. migratoria* CarE genes along with those known in other insect species. A) Alignment of deduced amino acid sequences of three *L. migratoria* CarE genes along with DmCG4382 and DmCG3841 in clade D. B) Alignment of deduced amino acid sequences of a *L. migratoria* CarE gene along with AgCOEjhe2E, AgCOEjhe3E and DmJHE in clade F. C) Alignment of deduced amino acid sequences of two *L. migratoria* CarE gene along with AgCOE7o and DmCG5397 in clade I. The multiple sequence alignment was performed using GeneDoc software. The predicted signal peptide is underlined in purple, the conserved motifs are boxed in red, N-terminal conserved Cys for disulfide bond is marked with purple star at the top, residues for oxyanion hole are linked with red line, the catalytic triads are marked with blue arrows at the top, and potential *N*-glycosylation sites are in orange.

**Figure 5:**
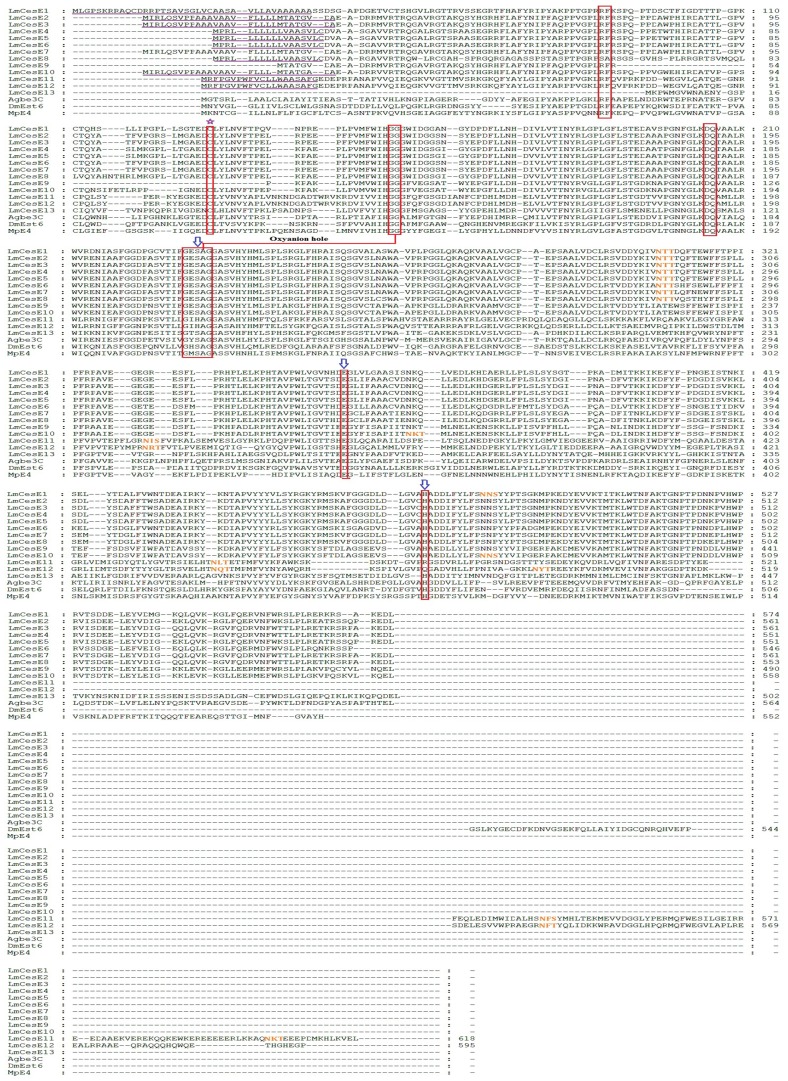
Alignment of deduced amino acid sequences of 13 *L. migratoria* CarE genes along with Agbe3C, DmEst6 and MpE4 in clade E. The multiple sequence alignment was performed using GeneDoc software. The amino acids of functional motifs are marked with asterisks. The predicted signal peptide is underlined in purple, the conserved motifs are boxed in red, N-terminal conserved Cys for disulfide bond is marked with purple star at the top, residues for oxyanion hole are linked with red line, the catalytic triads are marked with blue arrows at the top, and potential *N*-glycosylation sites are in orange.

The molecular characteristics of all the 39 CarEs are summarized in Tables 4 and 5. The calculated molecular mass for the 39 CarEs ranged from 52.2 to 92.6 KDa (Table 4). With the exception of four CarEs (pI 8.28 for LmCesA6, 8.18 for LmCesA7, 8.31 for LmCesA9 and 8.4 for LmCesI1), all the remaining CarEs are acidic or slightly acidic with isoelectric points ranging from 4.24 to 6.95. These characteristics of locust CarEs are consistent with those of previously known insect CarEs [34], [39]. The signal peptide cleavage sites were predicted in 28 locust CarEs, but absent in the remaining 11 CarEs (Table 4). Potential *N*-glycosylation sites were predicted to be presented in 28 locust CarEs (Figs. 3, 4 and 5).

**Table 4:** Predicated biochemical characteristics of 39 carboxylesterases deduced from *Locusta migratoria* cDNAs.

Gene name	GenBank accession number	Number of amino acid residues	Isoelectric point	Molecular mass (kDa)	Signal peptide and location
LmCesA1	JX961710	543	4.85	59.4	+(20−21)
LmCesA2	JX961711	501	4.37	55.8	+(20−21)
LmCesA3	KF849356	541	6.62	59.2	-
LmCesA4	KC986973	537	6.22	59.8	-
LmCesA5	KC986974	469	5.98	52.2	-
LmCesA6	KF849357	588	8.21	66.1	-
LmCesA7	KF849358	584	8.18	62.8	-
LmCesA8	KF849359	546	4.78	60.3	+(24−25)
LmCesA9	KF849360	544	8.31	60.3	-
LmCesA10	KF849361	545	4.32	59.5	+(19−20)
LmCesA11	KF849362	514	4.54	57.3	-
LmCesA12	KF849363	541	4.35	60.3	+(22−23)
LmCesA13	KF849364	546	4.38	60.8	+(22−23)
LmCesA14	KF849365	541	4.32	60.3	+(22−23)
LmCesA15	KF849366	546	4.35	60.8	+(22−23)
LmCesA16	KF849367	541	4.27	60.0	+(22−23)
LmCesA17	KF849368	546	4.27	60.6	+(22−23)
LmCesA18	KF849369	541	4.24	60.0	+(22−23)
LmCesA19	KF849370	546	4.29	60.6	+(22−23)
LmCesA20	KF849371	566	6.26	63.2	-
LmCesD1	KF849372	531	6.12	59.3	-
LmCesD2	KF849373	569	5.36	63.9	+(18−19)
LmCesD3	KF849374	570	6.54	63.7	+(30−31)
LmCesE1	KF849375	575	6.9	62.9	+(39−40)
LmCesE2	KF849376	561	5.85	62.4	+(29−30)
LmCesE3	KF849377	561	5.94	62.4	+(29−30)
LmCesE4	KF849378	551	6.29	60.9	+(17−18)
LmCesE5	KF849379	551	6.16	61.0	+(17−18)
LmCesE6	KF849380	546	5.89	60.5	+(17−18)
LmCesE7	KF849381	561	5.69	62.5	+(29−30)
LmCesE8	KF849382	553	6.95	60.9	+(17−18)
LmCesE9	KF849383	490	6.33	54.6	-
LmCesE10	KF849384	558	5.95	61.8	+(28−29)
LmCesE11	KF849385	618	5.96	70.3	+(20−21)
LmCesE12	KF849386	595	6.93	67.7	+(20−21)
LmCesE13	KF849387	502	5.95	56.2	-
LmCesF1	KF849388	830	4.79	92.6	+(22−23)
LmCesI1	KF849389	567	8.4	68.9	+(28−29)
LmCesI2	KF849390	602	6.23	65.6	+(19−20)

**Table 5:** Key amino acid residues (RF, DQ motifs, residues for nucleophilic elbow, catalytic triads, N-terminal conserved Cys for disulfide bond and oxyanion hole) of 39 carboxylesterases deduced from *Locusta migratoria* cDNAs.

Gene name	RF	DQ	Residues for nucleophilic elbow (GXSXG)	Catalytic triads	N-terminal conserved Cys for disulfide bond	Oxyanion hole
LmCesA1	+	+	GQSAG	S205E338H453	Cys100	G126G127A206
LmCesA2	-	+	GQSAG	S159E291H400	Cys53	G78G79A160
LmCesA3	+	+	GESAG	S208E341H459	Cys100	G127G128A209
LmCesA4	+	+	GESAG	S195E328H447	Cys87	G114G115A196
LmCesA5	-	+	GGSAG	S121E255H373	Cys14	G40G41A122
LmCesA6	+	+	GESAG	S234E369H489	Cys127	G153G154A235
LmCesA7	+	-	GQSAG	S213E347H479	Cys111	G134G135A214
LmCesA8	+	+	GESAG	S212E344H459	Cys104	G133G134A213
LmCesA9	+	+	GESAG	S195E328H447	Cys87	G114G115A196
LmCesA10	+	+	GESAG	S206D346H458	Cys103	G127G128A207
LmCesA11	+	+	GQSAG	S177E309H418	Cys64	D96G97A178
LmCesA12	+	+	GQSAG	S204E336H445	Cys98	G123G124A205
LmCesA13	+	+	GQSAG	S204E336H445	Cys98	G123G124A205
LmCesA14	+	+	GQSAG	S204E336H445	Cys98	G123G124A205
LmCesA15	+	+	GQSAG	S204E336H445	Cys98	G123G124A205
LmCesA16	+	+	GQSAG	S204E336H445	Cys98	G123G124A205
LmCesA17	+	+	GQSAG	S204E336H445	Cys98	G123G124A205
LmCesA18	+	+	GQSAG	S204E336H445	Cys98	G123G124A205
LmCesA19	+	+	GQSAG	S204E336H445	Cys98	G123G124A205
LmCesA20	-	+	GESAG	S190E323H466	Cys86	G109G110A191
LmCesD1	-	+	GYSAG	S164E294H412	Cys56	P83G84A165
LmCesD2	+	+	GYSVG	S211E343H462	Cys102	P130G131V212
LmCesD3	+	+	GYGAG	G220E350H470	Cys112	P139G140A221
LmCesE1	+	+	GESAG	S232E359H473	Cys129	G153G154A233
LmCesE2	+	+	GESAG	S217E344H458	Cys114	G138G139A218
LmCesE3	+	+	GESAG	S217E344H458	Cys114	G138G139A218
LmCesE4	+	+	GESAG	S207E334H448	Cys104	G128G129A208
LmCesE5	+	+	GESAG	S207E334H448	Cys104	G128G129A208
LmCesE6	+	+	GESAG	S207E334H448	Cys104	G128G129A208
LmCesE7	+	+	GESAG	S217E344H458	Cys114	G138G139A218
LmCesE8	-	+	GESAG	S209E336H450	Cys106	G130G131A210
LmCesE9	+	+	GESAG	S148E275H387	-	G69G70A149
LmCesE10	+	+	GESAG	S216E343H455	Cys113	G137G138A217
LmCesE11	+	+	GIHAG	H220E360R473	Cys108	G139G140A221
LmCesE12	+	+	GIHAG	H220E358Q472	Cys108	G139G140A221
LmCesE13	-	+	GTSAG	S143E272H394	Cys37	G64G65A144
LmCesF1	+	+	GEGAG	G303P434N554	Cys199	G222G223A304
LmCesI1	-	-	GQGTG	G235E377H509	Cys131	G156G157T236
LmCesI2	+	-	GGSAG	S222D366H475	Cys119	G143G144A223

The conserved motifs (RF, DQ, residues for nucleophilic elbow, catalytic triads, N-terminal conserved Cys, and oxyanion hole), necessary for maintenance of structural integrity of the molecule and the catalytic activity of CarEs and other esterases [40], were analyzed in the 39 locust CarEs (Table 5). For the RF motif, phenylalanine (F) was changed to tyrosine (Y) in LmCesA20 and to leucine (L) in LmCesI1; arginine (R) and phenylalanine (F) were substituted by serine (S) and alanine (A), respectively, in LmCesE8; phenylalanine (F) was missing in LmCesD1; and RF was missing in LmCesA2, LmCesA5 and LmCesE13. For the DQ motif, the substitution by DM was found in LmCesA7, LmCesI1 and LmCesI2. For the N-terminal conserved Cys, 38 locust CarEs retained this residue but LmCesE9 lacked this residue. Among the 39 CarEs, 34 displayed conserved motifs for catalytic triad (Ser-Glu/Asp-His; S-E/D-H). However, five including LmCesD3, LmCesE11, LmCesE12, LmCesF1 and LmCesI1 showed catalytic triads with at least one amino acid substitution. All these members lacked the serine residue for the catalytic triad (Table 5), and are therefore named CarE-like proteins in this study.

### Tissue-Dependent Expression Patterns of Four *L. migratoria* CarE Genes

**Figure 6:**
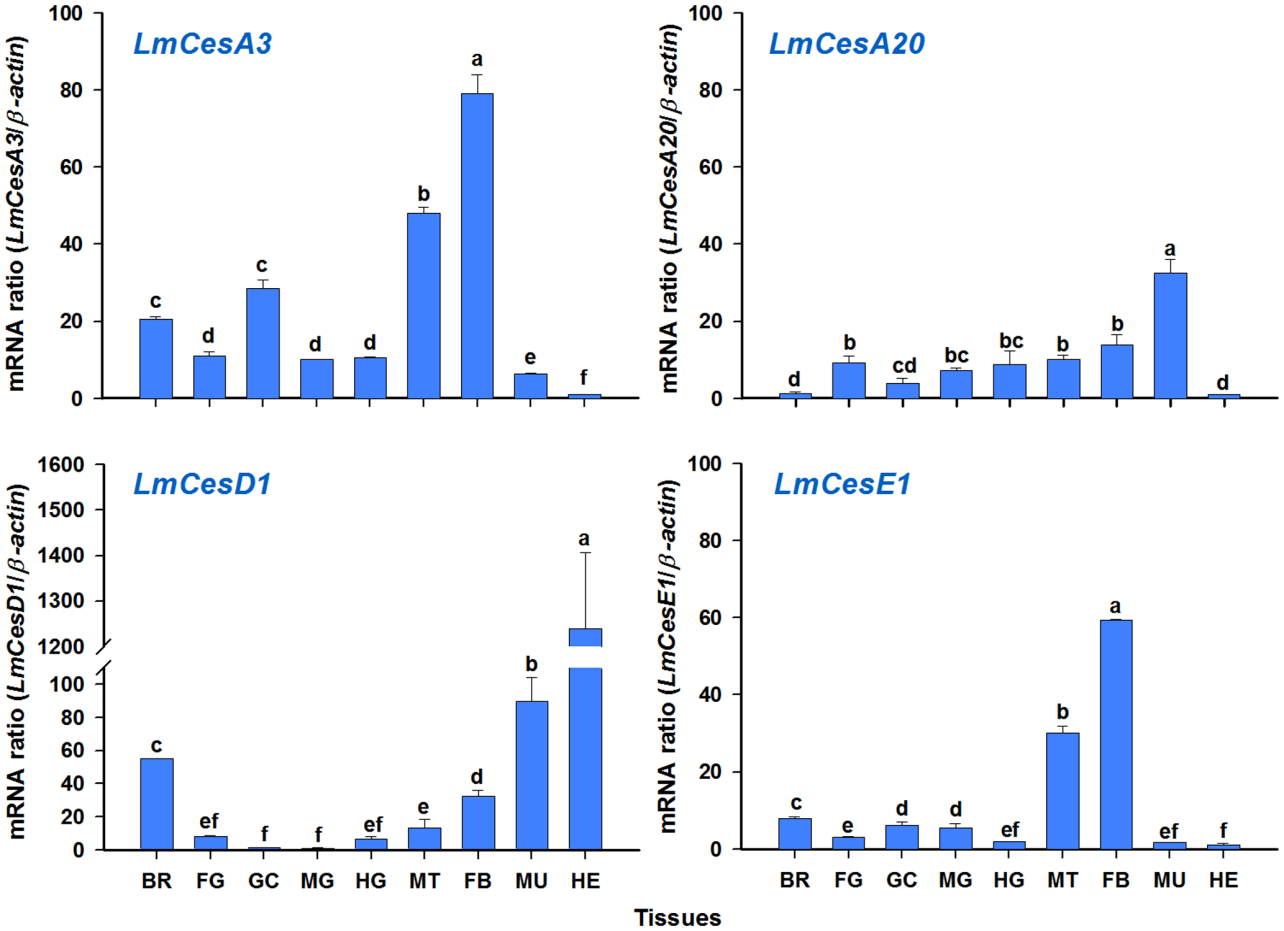
Tissue-dependent expression patterns of four CarE genes in *L. migratoria*. The analyses were conducted in nine different tissues, including the brain (BR), foregut (FG), gastric caeca (GC), midgut (MG), hindgut (HG), Malpighian tubules (MT), fat bodies (FB), muscle (MU) and hemolymph (HE). The mRNA level in each tissue was normalized by *β-actin* as a reference gene. Results are mean and standard errors (SE) of three biological replications (n = 3), each with three technical replications. Different letters above the vertical bars indicate significant differences among different tissues based on Fisher's LSD multiple comparison test (*P*<0.05).

We revealed the tissue-dependent expression patterns of the four CarE genes (*LmCesA3*, *LmCesA20*, *LmCesD1*, *LmCesE1*) in nine different tissues including the brain, foregut, gastric caeca, midgut, Malpighian tubules, hindgut, fat bodies, muscles and hemolymph by using RT-qPCR (Fig. 6). These genes were expressed in all the tissues examined, although there were significant differences in expression levels among different tissues. Specifically, for *LmCesA3* and *LmCesE1*, the highest expression was found in the fat bodies, followed by Malpighian tubules, but the lowest expression was found in the hemolymph. The highest expression of *LmCesA20* was found in the muscles, but the lowest expression was in the brain and hemolymph. In contrast, the highest expression of *LmCesD1* was observed in the hemolymph, followed by the muscles and brain, but the low expressions were found in the foregut, gastric caeca, midgut and hindgut.

### Stage-Dependent Expression Patterns of Four CarE Genes

**Figure 7:**
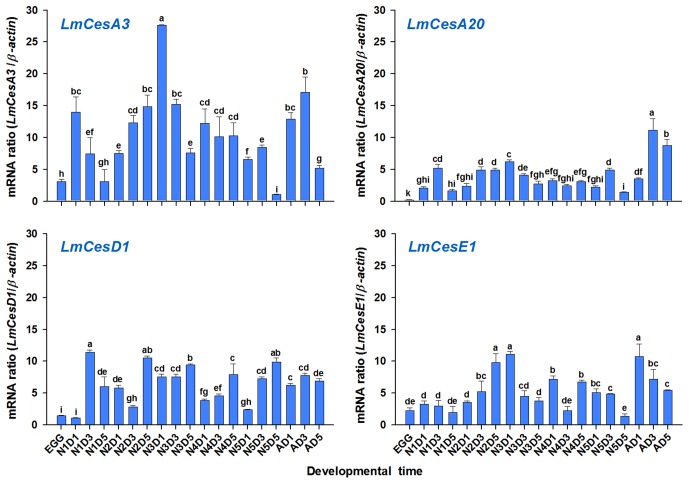
The relative expression levels of four CarE genes in different developmental stages of *L. migratoria* as evaluated by RT-qPCR. The mRNA level in each of the samples, including eggs (EGG), first-instar nymphs on day1, 3 and 5 (N1D1, N1D3 and N1D5), second-instar nymphs on day1, 3 and 5 (N2D1, N2D3 and N2D5), third-instar nymphs on day1, 3 and 5(N3D1, N3D3 and N3D5), fourth-instar nymphs on day1, 3 and 5 (N4D1, N4D3 and N4D5), fifth-instar nymphs on day1, 3 and 5 (N5D1, N5D3 and N5D5) and adults on day1, 3 and 5 (AD1, AD3 and AD5), was normalized by β-actin as a reference gene. Results are mean and standard errors (SE) of three biological replications (n = 3), each with three technical replications. Different letters above the vertical bars indicate significant differences among the samples, based on Fisher's LSD multiple comparison test (*P*<0.05).

The stage-dependent expression patterns of the four CarE genes in *L. migratoria* were analyzed by RT-qPCR (Fig. 7). The transcripts of all the four genes were detectable in all developmental stages, indicating that the expression of these four genes is ubiquitous in the locust. However, all the four genes showed relatively low expressions in eggs as compared with other developmental stages. In addition, there were significant variations in their expressions during the insect development.

### Effect of RNAi of Four CarE Genes on Susceptibility to Insecticides

**Figure 8:**
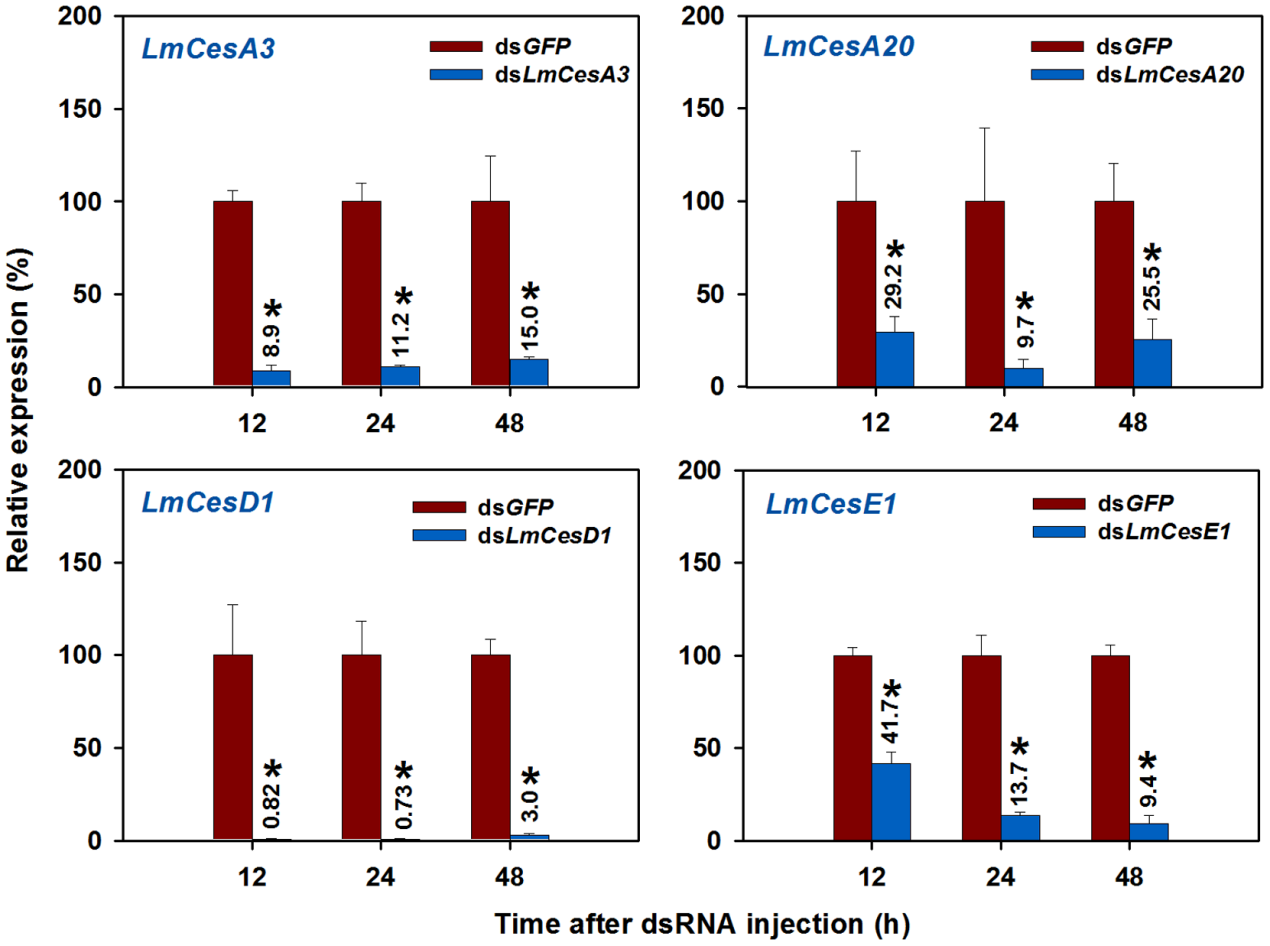
Efficiency of RNA interference for four *L. migratoria* CarE genes. The mRNA level was analyzed by **RT-qPCR** at three different time points after injection of 3 µg dsRNA (treatment) and normalized using *β-actin* as a reference gene (ds*GFP* as control).The dsGFP expression was set to 100%. Vertical bars indicate the standard errors (SE) of the mean (n = 3), whereas the symbol “*” indicates significant difference between the control and treatment in Student *t*-test (*P*<0.05).

Our RT-qPCR analyses showed significantly decreased transcript levels of each CarE target gene in the nymphs injected with an CarE gene-specific dsRNA (ds*LmCes*) as compared with the control insects injected with the dsRNA of the GFP gene (ds*GFP*) at three different time points (12, 24 and 48 h) after the dsRNA injections. The remaining transcript levels of the target genes were only <14% in the nymphs as compared with those in the controls at 24 h, indicating an effective silencing of these target genes by RNAi (Fig. 8).

**Figure 9:**
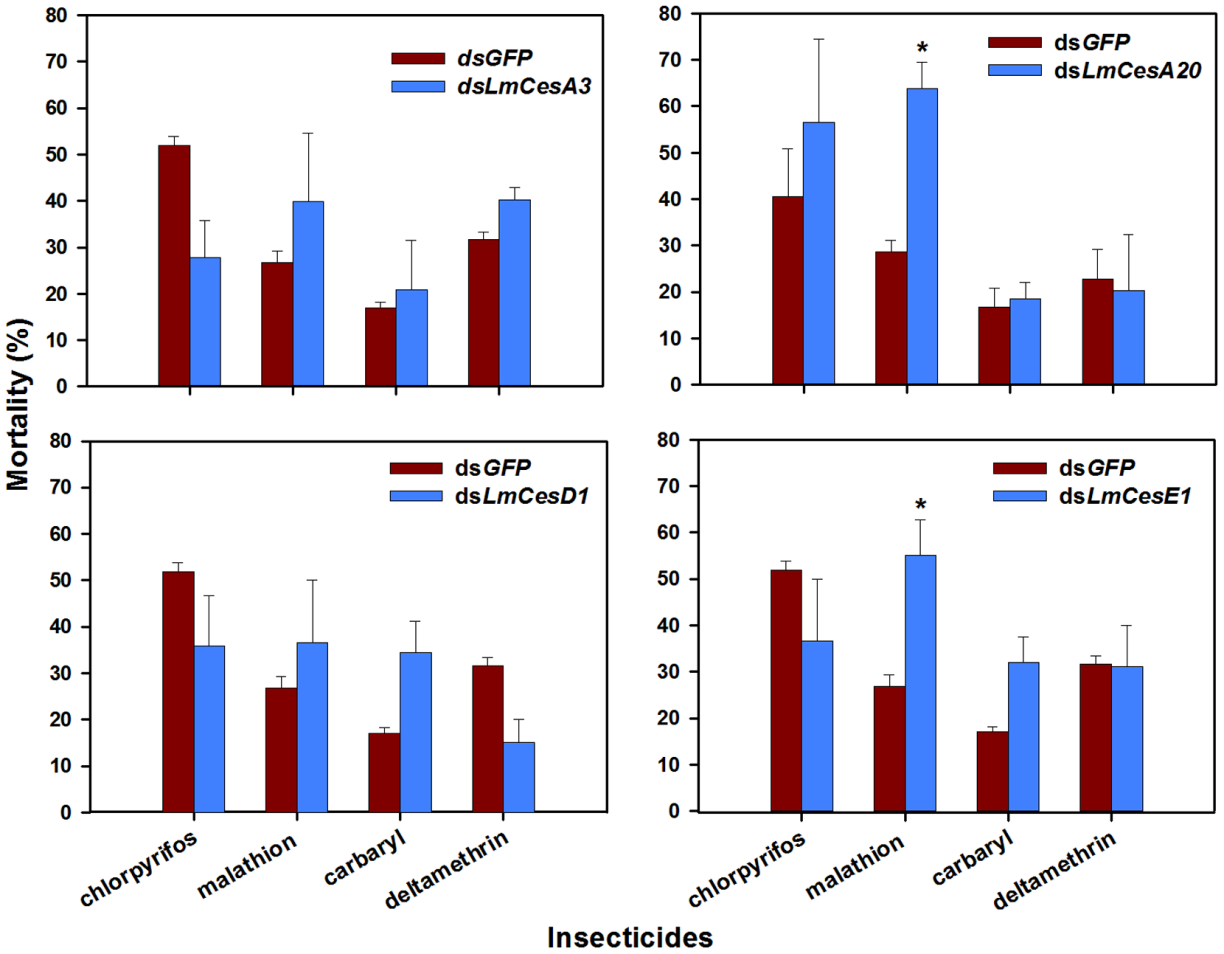
Changes in susceptibility of the locusts to different insecticides after the injection of *LmCesA3*, *LmCesA20*, *LmCesD1* or *LmCesE1* dsRNA in second instar nymphs. Insecticides bioassays were conducted 24(SE) of three independent experiments (n = 3). The symbol “*” indicates significant difference between the control and treatment in Student *t*-test (*P*<0.05).

As the transcript level of each CarE target gene was significantly repressed in the locusts by RNAi, we evaluated the susceptibility of the locusts to each of four insecticides (carbaryl, chlorpyrifos, deltamethrin and malathion). No mortality was observed in the nymphs injected with either ds*GFP* or any ds*LmCes* without insecticide exposures. However, the mortalities were 26.8, 63.9 and 55.2% in the nymphs injected with ds*GFP*, ds*LmCesA20* and ds*LmCesE1*, respectively, when the dsRNA injected locusts were exposed to malathion at the dose of LD_30_ (Fig. 9). These results represent 37.2 and 28.4% increases of the mortality in the nymphs injected with ds*LmCesA20* and ds*LmCesE1*, respectively, as compared with the nymphs injected with ds*GFP*. In contrast, the repression of either *LmCesA3* or *LmCesD1* transcript by RNAi did not significantly change the susceptibility of the locusts to malathion. Furthermore, the repression of each of the four CarE transcripts by RNAi did not significantly change the susceptibility of the locust to deltamethrin, chlorpyrifos or carbaryl.

## Discussion

Carboxylesterases (CarEs) belong to a superfamily of multifunctional enzymes associated with the degradation of endogenous and exogenous compounds including hormones, pheromones, drugs and insecticides [1], [2], [3]. In this study, we revealed 84 putative CarE cDNA sequences from the locust transcriptome database. This number was almost twice as many as the gene number (49) found in the genome of *A. gambiae*, but similar to that (71) found in *C. quinquefasciatus* [20] (Table 3). The number of CarEs in the neuro/developmental group was relatively conserved in dipteran (*D. melanogaster*, *A. gambiae*, *A. aegypti* and *C. p. quinquefasciatus*), hymenopteran (*N. vitripennis*, *A. mellifera*) and coleopteran (*T. castaneum*) insects [7], [20]. The numbers of CarEs in clades I, M and N show 1∶1∶1 ratios across the three genomes (Table 3). These results reflect the relatively ancient origin of this group, where all the members are catalytically inactive except for AChEs. Interestingly, we did not find any AChE cDNAs from the transcriptome database. The missing of the AChE cDNAs is probably due to their relatively short sequences in the database because we used the cutoff of 500 nucleotides in our searches. The missing of the AChE genes in our searches partially contributes to relatively few genes in the neuro/developmental group as identified in this study.

The number of CarEs in dietary/detoxification and hormone/semiochemical processing groups from the *L. migrotoria* transcriptome database exceeds what have been found in two dipteran insects (*D. melanogaster* and *A. gambiae*) [24] and a hemipteran insect (*T. vaporariorum*) [25]. Our comparisons of the known CarE genes in *L. migratoria*, *D. melanogaster*, *A. gambiae* and *T. vaporariorum* showed significant expansions of CarE genes in clade A (20 orthopteran α-esterase genes) of the dietary detoxification group and in clade E (13 β-esterase genes) of the hormone/semiochemical processing group in the locust (Table 3).

The dietary/detoxification group (clade A-C) is composed of α-esterases, which may be capable of detoxifying xenobiotics. Indeed, some members in this group are known to be associated with OP resistance in insects. For example, a hymenopteran CarE (AAC36246) that is involved in OP resistance belongs to this group [8]. In dipteran insects, the same amino acid substitution in CarEs confers OP resistance in both house fly and blow fly [41], [42]. In *B. mori*, Bmcce-5 with the GQSAG consensus motif is postulated to be involved in digestion or xenobiotic metabolism based on its expression patterns in Malpighian tubules and gut [10].

The in-depth studied clade E, which is the sister group to the nonlepidopteran JHEs, comprises secreted β-esterases from multiple insect species. Some members of β-esterases have well-described functions in several insect species. In *D. melanogaster*, Est6 (CG6917) plays a role in reproduction due to its high expression in ejaculatory duct [43], whereas Est7 (CG17148) is considered to be a functionless gene [44]. In addition, overexpressions of two esterase isozymes, E4 and FE4, have been reported to confer OP, carbamate and pyrethroid resistance in *M. persicae* [13], [15].

*A. mellifera* (honeybee) is a highly eusocial insect species and their reproductive individuals, in particular, are less likely to be exposed to environmental xenobiotics, such as insecticides. Thus, it is reasonable to expect relatively few detoxification genes in the reproductive individuals due to their specificjavascript:void(0); biological characteristics [22]. If the number of CarE genes reflects an insect's need for detoxification, why does the locust have such abundant detoxification genes compared with other insect species? Indeed, the migratory locust feeds extensively on various bulrush and gramineous plants, which contain many different plant secondary metabolites. Relatively frequent exposures of the insect to xenobiotics may be a part of the explanation for the expansion of the detoxification genes in the locust. Furthermore, our data support our hypothesis that the migratory locust as a serious agricultural pest is highly capable of developing insecticide resistance under the selection pressures in the field due to the presence of a large number of the detoxification genes.

CarEs are also known to play an important role in insect communications through the control of pheromone signal [45]. These esterases can inactivate the function of various pheromones by hydrolyzing their ester bonds [9]. The first sensillar esterase (Apol-SE) was found to be involved in pheromone degradation in the wild silkmoth *A. polyphemus* [45], [46]. More recently, a cDNA of a putative integumental esterase (Apol-IE, AAM14416) was sequenced in the same species [47]. In *D. melanogaster*, three integumental esterases have been identified, but in *A. gambiae* and *T. vaporariorum* none has been found (Table 3). Like in *D. melanogaster*, we found three integumental esterase genes (*LmCesD1*, *LmCesD2* and *LmCesD3*) in the locust transcriptome. These esterases share 40.7% identities with Apol-IE of *A. polyphemus* and 48.9% with EFA08586 of *T. castaneum* at the amino acid sequence level. These results suggest that these integumental esterases may be also involved in degrading pheromones in the locust.

The JH regulates the metamorphosis and reproduction in most insects [48], whereas the JHE is considered as a principal enzyme for hydrolyzing methyl ester of JH and changing JH titer in the hemolymph [49]. A variety of insects have only one physiologically functional JHE [38], [49]–[53]. In the locust, LmCesF1 was clustered with the nonlepidopteran JHE clade (F), and shares 28 and 33.6% amino acid identities with DmJHE (CG8424) and TmJHE (AAL41023.1), respectively. However, LmCesF1 and OxCesF (JHE-like protein of *Oxya*, unpublished) showed 52% identity. Interestingly, however, both LmCesF1 and OxCesF possess a modified catalytic triad (Gly-Pro-Asn) instead of Ser-Glu/Asp-His which has been found in typical CarEs. This suggests that both LmCesF1 and OxCesF are likely encoded by JHE-like genes, but may not be able to regulate the JH titer due to the change of their catalytic triads in these grasshopper species.

Indeed, the catalytic triad (Ser-Glu/Asp-His) is the basic feature of a CarE for its catalytic function. In *B. mori*, 15 α-estrerases show different amino acid residues in their catalytic triads, which may result in the loss of hydrolytic activity of these enzymes [23]. In our study, we found that most of the locust CarEs possessed a conserved catalytic triad. However, five CarEs (LmCesD3, LmCesE11, LmCesE12, LmCesF1 and LmCesI1) showed different amino acid substitutions of Ser-Glu/Asp-His (Table 5). It is reasonable to propose that these five genes may have lost their hydrolytic activity and perhaps acquired new functions in the locust. We also found possible *N*-glycosylation sites in 10 locust CarEs. *N*-glycosylation sites have been reported in mammalian CarEs [54], [55], and are considered to be necessary for secretory enzymes to maintain their stability and catalytic efficiency [56].

It has been suggested that different expression profiles of the genes encoding CarEs in different tissues may imply various roles of CarEs in mammalian and insect species [22], [55]. For example, *LmCesA3* and *LmCesE1* were predominantly expressed in the fat bodies and Malpighian tubules with the functions similar to those of the liver and kidney, respectively, in mammalian species. Because mammalian liver and kidney are known to play key roles in defending against toxic substances including insecticides and plant allelochemicals [57], [58], it is possible that LmCesA3 and LmCesE1 may have functions in metabolizing insecticides and allelochemicals in the locust.

We also found that *LmCesA3* was highly expressed in gastric caeca, which is an important organ for food absorption, water uptake and waste expulsion [59]. It is possible that LmCesA3 may be involved in the metabolism of chemicals in the food. Furthermore, three locust CarE genes (*LmCesA3*, *LmCesD1* and *LmCesE1*) were expressed in the brain. This expression pattern was similar to those of CES5 and CES6, which have been known to protect the brain and other neural tissues from damages caused by drugs in mammalian species [6], [60]. Therefore, LmCesA3, LmCesD1 and LmCesE1 may have the functions similar to those of CES5 and CES6. Specifically, these locust CarEs may help protect the insect brain and other neural tissues from damages caused by insecticides and other toxic substances.

Many insect CarEs have been known to play important roles in catalyzing the hydrolysis of a broad suite of ester-containing chemicals, such as OPs, carbamates and pyrethroids [61]. Malathion has been commonly used to control insect pests in the field because of its relatively low mammalian acute toxicity as compared with other OPs [62]. Like all OPs, however, malathion kills insects by irreversibly inhibiting AChE at cholinergic synapses [63]. Malathion, which is also an unusual OP with two carboxyl ester linkages in addition to the phosphoester linkage common to OPs, can be detoxified by malathion-specific CarEs (MCE) [4]. As validated by RNAi of *LmCesA20* and *LmCesE1* followed by insecticide bioassays, our results demonstrated that these two genes play an important role in malathion detoxification in the locust.

Chlorpyrifos is another widely used OP which can be detoxified by CarEs and other enzymes such as glutathione *S*-transferases [64]. Indeed, an increased CarE activity has been found to be responsible for chlorpyrifos resistance in *Bemisia tabaci* [65]. On the other hand, carbaryl is a commonly used carbamate insecticide and has been extensively applied in insect pest management programs. In the peach–potato aphid, elevated E4 activity has been known as a major mechanism conferring carbamate resistance. The E4 can hydrolyze carbaryl not only *in vivo* but also *in vitro* [66], [67]. On the other hand, deltamethrin can be hydrolyzed by the human liver CarEs, hCE-1 and hCE-2 [68]. Enhanced CarE activity has been implicated in deltamethrin resistance in various insect species [69], [70].

Nevertheless, LmCesA3, LmCesA20, LmCesD1 and LmCesE1 don’t seem to play any important role in detoxification of chlorpyrifos, carbaryl and deltamethrin as silencing each of these four genes did not increase the susceptibility of the locust to these insecticides. Our results suggest that the role of different CarEs in insecticide detoxification is not only gene specific but also insecticide specific in the locust.

## Conclusion

In this study, we identified 39 CarE cDNAs with complete ORFs from the *L. migratoria* transcriptome database. The deduced proteins of these CarE cDNAs were clustered into five different clades in our phylogenetic analysis. Significant expansions of the CarE genes were found in clade A (20 orthopteran α-esterase genes) of the dietary detoxification group and in clade E (13 β-esterase genes) of the hormone/semiochemical processing group. Among the four CarE genes (*LmCesA3*, *LmCesA20*, *LmCesD1* and *LmCesE1*) selected for further analyses, *LmCesA20* and *LmCesE1* appeared to play an important role in detoxification of malathion but not in detoxification of chlorpyrifos, carbaryl and deltamethrin. Furthermore, none of the four genes appeared to play any significant role in detoxification of these three insecticides. Our study suggests that the detoxification function of CarEs is both gene and insecticide specific. These results are expected to help researchers reveal the characteristics of diverse CarEs and assess the risk of insecticide resistance conferred by CarEs in the locust and other insect species.

## Supporting Information

Figure S1 **Phylogenetic analyses of insect carboxylesterases (CarEs).** MEGA 5 was used to construct the phylogenetic tree with neighbor-joining method. Nodes with distance bootstrap values greater than 50% (1000 replicates) are shown. The nomenclatures of the clades are according to Oakeshott et al [4]. The 71 deduced CarEs (including 32 CarEs fragments and 39 CarEs with complete ORF) from *L. migratoria* are marked with red triangle. The accession numbers for various CarEs of insects used in this analysis were generally taken from NCBI database (http://www.ncbi.nlm.nih.gov/). The tree included twelve clades named A-N (except for C and J clade). The abbreviations used for insect species are: *Drosophila melanogaster* (Dm), *Anopheles gambiae* (Ag), *Culex quinquefasciatus* (Cq), *Aedes aegypti* (Aa), *Lucilia cuprina* (Lc), *Haematobia irritans* (Hi), *Musca domestica* (Md), *Myzus persicae* (Mp), *Antheraea polyphemus* (Ap), *Manduca sexta* (Ms), *Choristoneura fumiferana* (Cf), *Bombyx mori* (Bm), and *Tribolium castaneum* (Tc).
(TIF)
