# Supplementary figures and images for: Molecular and Functional Characterization of cDNAs Putatively Encoding Carboxylesterases from the Migratory Locust, Locusta migratoria
Source: PLoS One. 2014 Apr 10;9(4):e94809. doi: 10.1371/journal.pone.0094809 (PMC3983256; doi:10.1371/journal.pone.0094809)

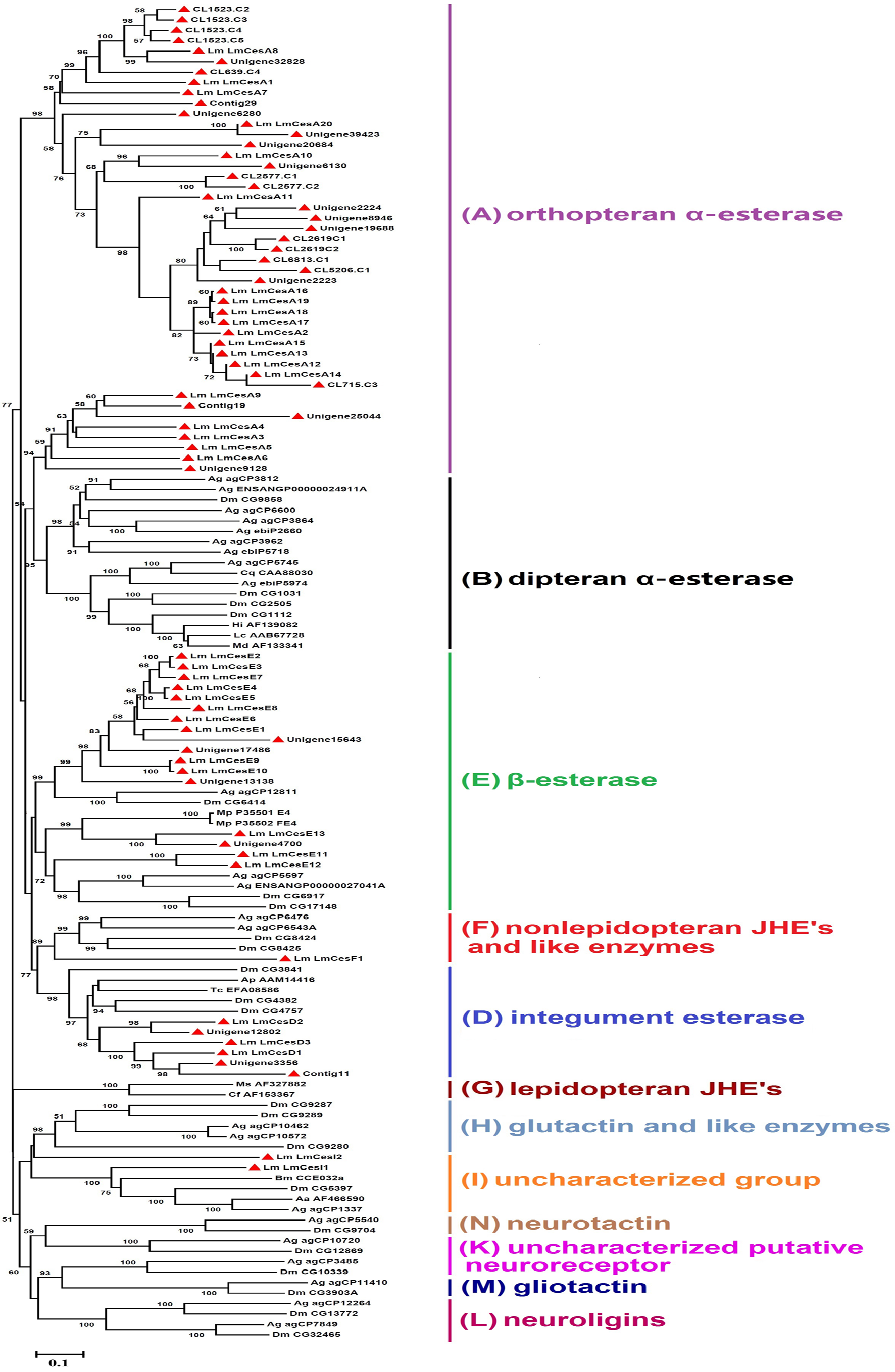

Supplement: Figure S1 — Phylogenetic analyses of insect carboxylesterases (CarEs). MEGA 5 was used to construct the phylogenetic tree with neighbor-joining method. Nodes with distance bootstrap values greater than 50% (1000 replicates) are shown. The nomenclatures of the clades are according to Oakeshott et al [4]. The 71 deduced CarEs (including 32 CarEs fragments and 39 CarEs with complete ORF) from L. migratoria are marked with red triangle. The accession numbers for various CarEs of insects used in this analysis were generally taken from NCBI database (http://www.ncbi.nlm.nih.gov/). The tree included twelve clades named A-N (except for C and J clade). The abbreviations used for insect species are: Drosophila melanogaster (Dm), Anopheles gambiae (Ag), Culex quinquefasciatus (Cq), Aedes aegypti (Aa), Lucilia cuprina (Lc), Haematobia irritans (Hi), Musca domestica (Md), Myzus persicae (Mp), Antheraea polyphemus (Ap), Manduca sexta (Ms), Choristoneura fumiferana (Cf), Bombyx mori (Bm), and Tribolium castaneum (Tc). (TIF) [file pone.0094809.s001.tif]
